# Whirlpool No More: A Case of Misdiagnosed Malrotation with Midgut Volvulus

**DOI:** 10.5811/cpcem.2021.9.52682

**Published:** 2021-10-28

**Authors:** Michael Fogam, Natasha Leigh, Trent She

**Affiliations:** * Mount Sinai St. Luke’s and Mount Sinai West, Department of Emergency Medicine, New York, New York; † Mount Sinai St. Luke’s and Mount Sinai West, Department of Surgery, New York, New York

**Keywords:** malrotation, midgut volvulus, whirlpool sign

## Abstract

**Introduction:**

Adult intestinal malrotation with midgut volvulus is rare and most often diagnosed on abdominal imaging. Once the diagnosis is made, prompt surgical intervention is necessary. A finding suggestive of malrotation with midgut volvulus on abdominal imaging is the “whirlpool” sign where the superior mesenteric vein and superior mesenteric artery twist at the root of the abdominal mesentery. This sign was once thought to be pathognomonic, but recent studies have shown that it can be seen in asymptomatic patients.

**Case Report:**

A 20-year-old female presented to our emergency department with diffuse abdominal pain. Computed tomography demonstrated the “whirlpool” sign with a concern for malrotation with midgut volvulus. Surgical consultation was obtained and the patient was rushed to the operating room for an exploratory laparotomy. Normal mesenteric attachments were seen and no significant pathology was identified during the laparotomy. The patient was eventually diagnosed with gastritis and discharged in stable condition.

**Conclusion:**

Emergency physicians and surgeons alike should be cautious in confirming malrotation with midgut volvulus solely due to the “whirlpool” sign on abdominal imaging. Premature diagnostic closure can lead to unnecessary procedures and interventions for patients as in the case we report here.

## INTRODUCTION

Malrotation with midgut volvulus is an uncommon surgical emergency in adults. The diagnosis is established with certain characteristic findings on computed tomography of the abdomen and pelvis (CT A/P). The most notable finding is a counterclockwise twisting of the superior mesenteric vein (SMV) onto the superior mesenteric artery (SMA), also known as the “whirlpool” sign.^1^ However, because this finding can sometimes be a normal variant care must be exercised to correlate the “whirlpool” sign with clinical examination to prevent unnecessary intervention.

## CASE REPORT

Our patient was a 20-year-old female who presented to the emergency department (ED) with two hours of diffuse abdominal pain. She had similar episodes of abdominal pain in the past that occurred once or twice a year after food intake, which were relieved with pain medication. On current presentation, her pain was far worse than usual.

Initial vitals were unremarkable, without tachycardia and without fever. Upon examination, the patient’s abdomen was soft but diffusely tender from the epigastrium to the hypogastrium. She exhibited no guarding or rebound tenderness. Her laboratory studies included an unremarkable chemistry panel, a slight leukocytosis of 11.3 thousand cells per microliter (K/μL) (reference range: 3.9 – 11 K/μL), and a normal serum lactate of 0.76 millimoles per liter (mmol/L) (normal < 2 mmol/L). An upright chest radiograph did not demonstrate pneumoperitoneum. Intravenous morphine and famotidine were administered with minimal relief of symptoms. Computed tomography of the abdomen and pelvis (CT A/P) with oral and intravenous contrast demonstrated a distended stomach, a duodenum that did not definitively cross midline, mildly dilated small bowel loops that were mostly in the right lower quadrant and, most distinctively, an SMV that twisted in a 360° counterclockwise fashion to the left of the SMA. A presumed diagnosis of malrotation and midgut volvulus was made and prompt surgical consultation was obtained.

The patient was taken emergently to the operating room for an exploratory laparotomy. The bowel was found to be in its proper anatomic position with normal peritoneal attachments and without the presence of any Ladd’s bands. (These fibrous bands of peritoneal tissue are embryologic remnants and the ultimate cause of malrotation.) On postoperative day one the patient underwent an upper endoscopy, revealing erosions and erythema in the stomach and superficial duodenal ulcers. Her postoperative recovery was unremarkable, and she was discharged home uneventfully with the diagnosis of gastritis and peptic ulcer disease.

## DISCUSSION

Malrotation is a condition resulting from an embryologic abnormality: failure of the bowel to rotate in a 270° counterclockwise fashion and fixate on the posterior abdominal wall. Without this fixation, the small bowel can twist about the base of its mesentery onto the large bowel, resulting in a midgut volvulus. Classically, this condition presents in infancy, with 90% of cases occurring under the age of one. Only 0.2–0.5% of cases of intestinal malrotation occur in adulthood, and of these cases only 15% present with a midgut volvulus.^1^ Traditionally, an upper gastrointestinal (GI) series has been the imaging modality of choice demonstrating a classic “corkscrew” appearance when contrast does not pass through the duodenum.^2^ In adults, CT A/P is much more commonly used.^3^

**Image 1:**
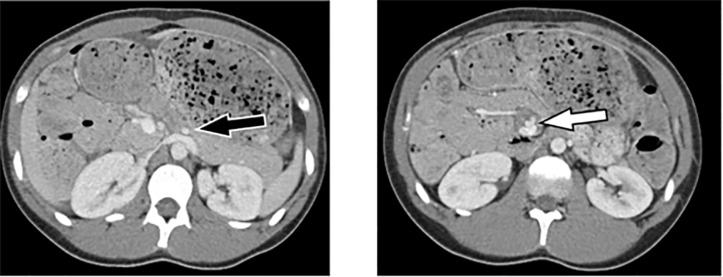
Left-hand image showing branch point of the superior mesenteric artery from the abdominal aorta on axial computed tomography views (black arrow). Right-hand image showing “whirlpool” sign (white arrow).

**Image 2:**
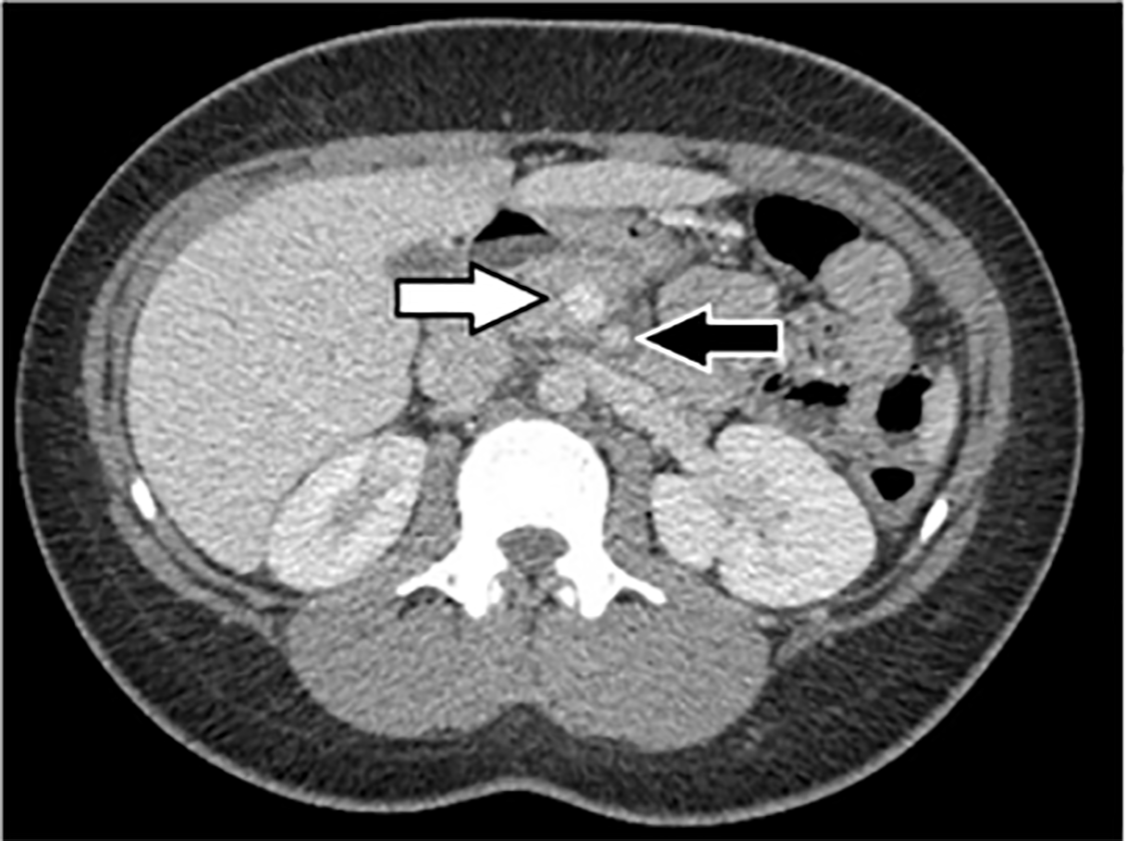
This axial computed tomography image shows the normal appearance of the superior mesenteric artery (black arrow) and just adjacent to it, the superior mesenteric vein (white arrow).

A finding previously considered to be pathognomonic^1^ for midgut volvulus is the “whirlpool” sign (Image 1), or the counterclockwise twisting of the superior mesenteric vein on the superior mesenteric artery as the small bowel mesentery twists about its axis. More recent research suggests that although this sign may be suggestive of midgut volvulus, it is not diagnostically definitive. Although the SMV normally runs concurrently with the SMA in close proximity (Image 2), 10–36% of the pediatric population have SMV/SMA twisting at baseline,^4^–^5^ and up to 33% of these have a 270° or greater twist.^5^ Some coexisting intra-abdominal findings such as renal masses or ascites can be present, but often the reason for this asymptomatic variance is unclear.^6^

**CPC-EM Capsule**

What do we already know about this clinical entity?

*Malrotation with midgut volvulus is a condition requiring timely diagnosis in the emergency department and may be present at any age. It should not be thought of as a strictly pediatric problem.*

What makes this presentation of disease reportable?

*Malrotation with midgut volvulus will be suggested by findings on computed tomography including a counterclockwise twisting of the superior mesenteric vein on the superior mesenteric artery.*

What is the major learning point?

*Caution must be used when interpreting the above “whirlpool” sign as it is not necessarily pathognomonic of malrotation as was previously thought.*

How might this improve emergency medicine practice?

*Emergency physicians should not just rely on the finding of the “whirlpool” sign to diagnose malrotation and should consider all clinical and radiographic data available.*

Our patient ultimately did not have malrotation with midgut volvulus despite radiographic findings suggestive of malrotation including a positive “whirlpool” sign, gastric distention, and most of the small bowel loops in the right lower quadrant. She did not have any laboratory findings suggestive of ischemia.

Her presentation was suggestive of malrotation but can also be explained by peptic ulcer disease or other upper gastrointestinal causes of pain. Our patient’s case illustrates the point that a “whirlpool” sign is not in itself pathognomonic for malrotation and can be a normal variant, and that clinical correlation is key to determining indications for operative intervention. Rather than surgical intervention in this case, continued observation with serial abdominal exams with possible endoscopic intervention or an upper GI series could have been pursued.

## CONCLUSION

Malrotation with midgut volvulus is an important condition requiring timely diagnosis in the ED and may be present in all age groups. It should not be thought of as a strictly pediatric problem. Very often, the diagnosis will be suggested by findings on a CT of the abdomen/pelvis including a counterclockwise twisting of the superior mesenteric vein on the superior mesenteric artery. Caution must be used when interpreting this “whirlpool” sign, as it is not necessarily pathognomonic of malrotation as was previously thought.
